# Continued Advancement in Understanding of Corticosterone in Psittacines

**DOI:** 10.3390/ani16142160

**Published:** 2026-07-11

**Authors:** Timothy J. Brunner, Hailey B. Penticoff, Thomas N. Tully

**Affiliations:** Department of Veterinary Clinical Sciences, School of Veterinary Medicine, Louisiana State University, 1909 Skip Bertman Drive, Baton Rouge, LA 70803, USA; tbrunner@lsu.edu (T.J.B.); hpenticoff@lsu.edu (H.B.P.)

**Keywords:** parrots, avian, corticosterone, diagnostic testing, stress, bird

## Abstract

Understanding physiology and the manner in which psittacine and other avian species cope with stress, as well as the physiological and psychological effects of acute and chronic stress on their body, is critical in learning how to best care for and manage these populations, both in captivity and in the wild. The way birds respond to and cope with both acute and chronic stress plays an important role in several physiological and metabolic functions, including homeostasis, glucose mobilization, activation of the sympathetic and parasympathetic nervous system, and the fear response. Corticosterone is a glucocorticoid that regulates secretion along the hypothalamic–pituitary–adrenal axis and mediates neurotransmission and humoral regulation. There are different methods used in measuring corticosterone concentrations in birds. It is necessary for the reader to understand these methods when assessing the results and conclusions in a published study. Along with understanding the samples to be submitted and the time periods they represent, it is also important to consider what factors have been identified to cause variation in corticosterone concentrations. Many of the avian research studies involving corticosterone have looked at measurements related to how real or perceived stressors affect the overall condition of the bird. The overall condition of the bird may be evaluated as feather quality, immune status, quality of life, metabolic and reproductive activity, and/or response to a typical veterinary clinical setting.

## Simple Summary

There are over 11,000 species of birds in the world. Many of these avian species are endangered, maintained in zoological parks, and are commonly maintained as companion animals. In all situations, birds are confronted with stressors throughout their life. In birds, corticosterone is the primary glucocorticoid and is measured to evaluate a bird’s response to stressors. There has been a paucity of published information regarding corticosterone concentrations in response to natural or simulated stressors in avian species. Currently, there is an expanding body of work that is focusing on this subject to obtain a better understanding of stress and its effects on avian species. With this new information and advances in measuring corticosterone concentrations in birds, we provide a review of corticosterone and how this hormone physiologically reacts to stress and the influence it has on the overall health of the animal. To determine the corticosterone level in a bird, a method to measure this steroid hormone is required. The different methods to measure corticosterone are assessed and described for both their positive qualities and limitations. Through the description of corticosterone, its response to stressors, both acute and chronic, and a review of recent research activity, the information within provides one with a better understanding of this hormone while offering recommendations for future research opportunities.

## 1. Introduction

Psittacines comprise a diverse group of birds that veterinarians have contact with in various settings, including their native ecological range, zoos, or as companion birds the world over. One-third of all known parrot species are classified as at-risk populations [1]. Therefore, an increased understanding of physiology and the way birds cope with stress, as well as the physiological and psychological effects of acute and chronic stress on their body is critical in learning how to best care for and manage avian populations. This review aims to discuss how corticosterone (CORT) is synthesized and its related anatomy, the effects CORT has on various body systems, suitable samples for collection, and variables that affect the secretion of CORT. We then review current research and future directions for research into CORT.

**Figure 1:**
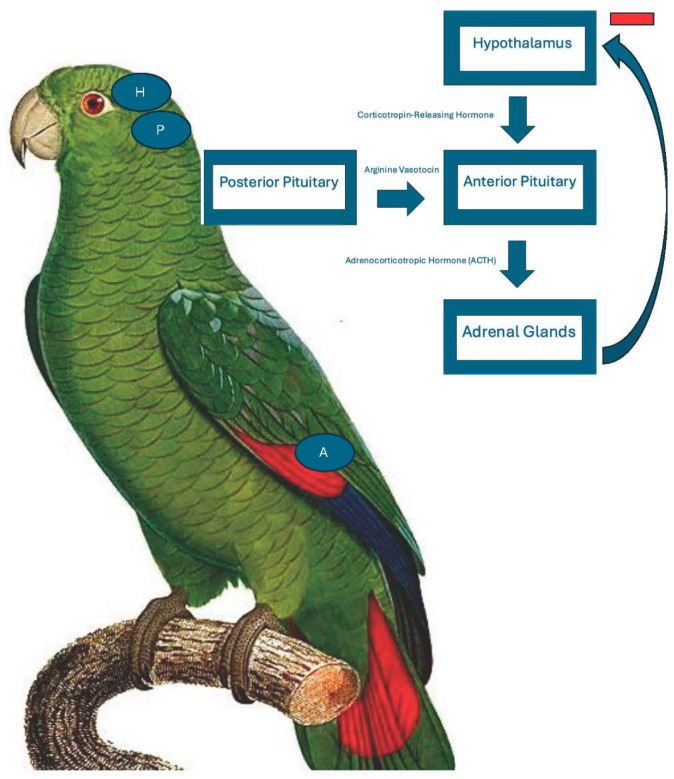
Hypothalamic–pituitary–adrenal (HPA) axis in a black-billed amazon (*Amazona agilis*). This image demonstrates the HPA axis and the hormones involved in the stress response, as well as the negative feedback loop. H = hypothalamus. P = pituitary. A = adrenal glands. The red bar symbolizes negative feedback.

The manner in which birds respond to and cope with both acute and chronic stress plays an important role in several physiological and metabolic functions, including homeostasis, glucose mobilization, activation of the sympathetic and parasympathetic nervous system, and the fear response [2,3]. Stress is defined as a state in which the hypothalamo–pituitary–adrenal (HPA) axis is activated with increased secretion of glucocorticoids in response to a stressor (Figure 1) [3]. The secretion of glucocorticoids is an important mechanism for an animal to respond to a stressful situation or condition. Moreover, glucocorticoids are a commonly used biomarker to assess welfare and overall stress in several other taxa [4]. A stimulus can be physical or emotional and is only considered a stressor if it activates the HPA axis [3]. Physical stressors involve signals arising from the body with a disturbance involving physical or chemical tissue effects. In mammals, emotional stressors require the appraisal of information in relation to stored information that is learned or inherited and must be processed in the limbic system or cortex of the brain (i.e., the sight of a predator) [3]. Glucocorticoids are steroid hormones that are produced in the adrenal gland cortex and have a primary role in glucose, protein, and fat metabolism. Additionally, these steroid hormones play a significant role in anti-inflammatory and immunosuppressive processes [5]. In a non-stressed, undisturbed animal, the glucocorticoid secretion is relatively low, which may fluctuate with circadian and seasonal rhythm along with metabolic demands to maintain homeostasis [6].

Corticosterone is the primary glucocorticoid in birds [7]. This differs from mammals, in which the primary glucocorticoid is cortisol [8]. Corticosterone is a glucocorticoid that regulates secretion after activation of the hypothalamic–pituitary–adrenal (HPA) axis and mediates neurotransmission and humoral regulation. The secretion of CORT is stimulated by adrenocorticotropic hormone (ACTH) from the pituitary gland, which is stimulated by corticotropin-releasing factor and arginine vasotocin from the hypothalamus [3]. After the HPA axis is activated, an increase in plasma CORT is detectable in plasma after 1–2 min following the initial stressor [9]. When the bird is undergoing a stressful event, CORT signals the activation of the immune system. Depending on the degree and duration of stress, CORT concentrations will fluctuate; therefore, its effects on the immune system will also vary [5]. The systemic effects of corticosterone are widespread due to the widespread presence of glucocorticoid receptors throughout the body [5].

The level of response to stress varies widely among species and individuals within a species [3], especially when considering companion birds, those maintained in exhibits (e.g., zoo), and those in the wild. Generally, domesticated birds (e.g., poultry) have a lower CORT response compared to free-ranging birds [10]. Captive birds that are free-living in an aviary or similar setting have a similar CORT response to those in the wild, as observed in captive great tits (*Parus major*) and wild willow tits (*Poecile montanus*) [11,12]. Psittacines live in a variety of environments, including in homes as companion birds, free-living in aviaries, or in their natural habitats. As such, it is important to consider the bird’s environment and daily stimuli and stressors to which it is exposed. As such, captive birds have different energetic demands compared to those in the wild, as there is a decreased need to travel long distances to forage, build nests, and raise young [13].

At normal (baseline) concentrations, CORT and similar glucocorticoids in other species have primary metabolic functions, including managing energy intake, storage, and mobilization through the metabolism of proteins and lipids into carbohydrates [3,13]. In the acute phase after exposure to a stressor, the secretion of CORT leads to the mobilization of glucose, changes in behavior, and the activation of the sympathetic nervous system and immune system through inflammation [3,5]. The secretion of CORT triggers a negative feedback response in the HPA axis, which in turn inhibits CORT secretion [5]. Long-term, repeated stress followed by the negative feedback from the HPA axis results in feedback failure, in which the physiological response is gradual elevations in baseline CORT concentrations that cannot be reversed [5]. The repeated secretion of CORT in animals has been shown to lead to HPA axis dysfunction, neuronal damage, cognitive decline, and memory impairment [5]. Behaviorally, acute stress and subsequent upregulation of CORT are associated with foraging and escape behaviors [3]. Long-term (chronic) stress and continued dysregulation of the HPA axis and elevated baseline CORT concentrations lead to an increased immune response, resulting in long-term inflammation throughout the body through continuous production of inflammatory cytokines [5]. Behaviorally, this has been associated with reduced nestling growth rates, reduced nestling body condition, increased begging rates in nestlings, and immunosuppression [14]. Chronic elevations of CORT in the nestling stage can also have lasting effects well into adulthood, including effects on feather length and quality, song quality and dominance rank in other species [14]. Therefore, reducing chronic stress in vulnerable populations is important in maintaining normal mating/nesting behavior as well as ensuring the appropriate maturation of nestlings. There are variables that affect baseline CORT concentrations, including age, sex, and seasonality, that have been and are currently being investigated.

There are several aspects of veterinary medicine that are known to be stressful and induce the activation of the HPA axis in psittacines. Restraint for physical examinations and performing routine diagnostic tests, such as phlebotomy, has been shown to lead to an increase in CORT [2,15]. It has been demonstrated that avian plasma CORT concentrations are detectable within 1–2 min after initial exposure to a stressor, with peak concentrations reached at 15–30 min post-exposure and returning to baseline 60–90 min after the stressor has been removed [9]. The release of CORT and other endogenous corticosteroids and catecholamines associated with stress is known to affect several physiological and biochemical parameters in the body and should be considered when interpreting clinical data [16]. One such example in mammals is the “stress leukogram” that has been described in domestic cats and dogs, where stress leads to an increase in segmented neutrophils and monocytes, and a decrease in lymphocytes and eosinophils on a white blood cell differential count [17]. A study conducted in 2018 showed a significant difference in the ratio of heterophils to lymphocytes in birds that were restrained for transport, a physical examination, and grooming to simulate stress, compared to baseline heterophil/lymphocyte ratios when blood was collected in under 3 min [16]. The biomedical technological advances in the ability to measure plasma corticosterone were essential to investigating “real-time” CORT concentrations in birds, as was the case in the aforementioned investigation. There are different methods used in measuring CORT concentrations in birds. It is necessary for the reader to understand these methods when assessing the results and conclusions in a published study.

## 2. Methods of Measuring Corticosterone Concentrations in Birds

### 2.1. Blood Corticosterone

Blood CORT, or more commonly plasma CORT, is the most well-studied sample as it relates to psittacines. In Hispaniolan Amazon parrots (*Amazona ventralis*), it has been noted that within 20 min of handling, the corticosterone concentrations will increase as compared to their baseline. Even with prolonged handling, the level of CORT seems to plateau after this initial increase [16]. However, this was in captive animals. In a separate study comparing captive and wild birds, captive-bred parrots had concentrations of CORT that would decrease by 45 min after prolonged handling, whereas they continually increased in wild parrots [15]. Just as important as prolonged handling is the time it takes for CORT concentrations to increase. It has been established that samples need to be collected within 3 min, as after this time, the “baseline” values will be artificially elevated from handling [9].

**Figure 2:**
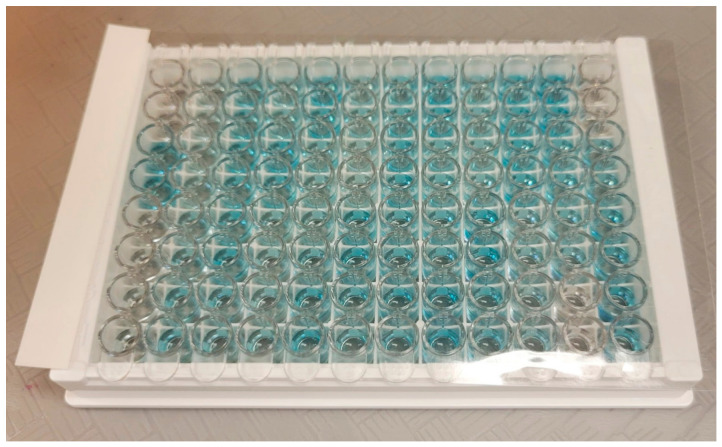
Corticosterone Multi-Format enzyme-linked immunosorbent assay (ELISA) plate from Arbor Assays K014-H Corticosterone Multi-Format ELISA Kit (Arbor Assays, Ann Arbor, MI, USA) is used with confidence by researchers investigating corticosterone levels in avian species.

While specific research is needed for birds, the method in which the plasma corticosterone is measured should all be considered. In mammals, radioimmunoassay is more commonly used and thought to be a better measure of CORT than enzyme-linked immunosorbent assay (ELISA) in mammals [18]. Conversely, in birds, the ELISA measurement of CORT is used with confidence and support of prominent avian researchers, in which avian CORT is a focus of their laboratory (Figure 2). To obtain the best result, it is important to be consistent in the test being used if serial samples are being submitted.

### 2.2. Feather Corticosterone

Feather CORT has also gained attention as a non-invasive way to measure corticosterone over time. As a feather grows and is connected to its blood supply, CORT is deposited in the keratin. This mechanism explains why feather CORT is a better reflection of a longer time period, often days to weeks, depending on the species of bird [19]. However, this also explains the limits of measuring feather CORT; it is only reflective of CORT concentrations during the time of feather growth [20]. Moreover, due to the stress associated with feather plucking, a recent study in domestic geese (*Anser anser domesticus*) and the mallard ducks (*Anas sterilis*) demonstrated that cut feathers can also be used as an alternative sample [19].

While studies have been performed looking at the relationship between feather-destructive behavior and plasma CORT in psittacines, few studies exist on feather CORT in these species [21]. One such study, investigating wild rainbow lorikeets (*Trichoglossus moluccanus*) in a wildlife rehabilitation center, sought to compare feather CORT between patients presenting for acute and chronic injuries [22]. Further studies are required in psittacine species, especially those with feather-destructive behavior, to test their reliability and usefulness.

### 2.3. Fecal Corticosterone

Fecal CORT has been utilized in a variety of avian orders, including California spotted owls (*Strix occidentalis occidentalis*), black grouse (*Lyrurus tetrix*), and Carolina chickadees (*Poecile carolinensis*) [23,24,25]. Specific to psittacines, it has been used in African gray parrots and peach-faced lovebirds (*Agapornis roseicollis*) [21,26]. However, it is important to note that in addition to CORT, depending on the assay and the specificity, there may be cross reactivity with other glucocorticoids, such as desoxycorticosterone, tetrhydrocorticosterone, aldosterone and primarily cortisol [13].

Beyond the different metabolites that may appear in the fecal test, fecal CORT reflects metabolized CORT from the blood. Therefore, it is not a single time point, as it is with plasma values. Instead, it is more of an “average” over a certain time point, depending on the species. Previous studies have indicated that it can take between 2 and 24 h for an increase in plasma corticosterone to be reflected in the metabolites in the feces [27]. This also provides a limitation, as steroids, including CORT, need to be metabolized in the liver and excreted in the feces. Therefore, depending on the bird’s health status and gastrointestinal transit time, there is expected to be species variation [28].

### 2.4. Variations

Along with understanding the samples to be submitted and the time periods they represent, it is also important to consider what factors have been identified to cause variation in CORT. It has been proven across other avian orders, especially in passerines, that there is a diel fluctuation in CORT [29]. Corticosterone will be highest in the morning, often at the beginning of a diurnal species activity period. While no such studies exist for plasma CORT in psittacines, a study in blue-fronted parrots (*Amazona aestiva*) demonstrated that fecal glucocorticoids peaked in the morning hours and were lowest in the evening [30]. Further research is needed for psittacines, especially regarding the effects of the circadian vs diel rhythms, as well as the differences between equatorial and temperate species.

Beyond fluctuations throughout the day, previous studies have sought to explore the effects of sex on CORT concentrations. While several studies have demonstrated that baseline plasma CORT concentrations do vary between sexes, the change in CORT concentrations with handling varies based on species. In Puerto Rican parrots (*Amazona vittata*), males will have a higher level of CORT after 30 min of restraint [13]. However, in Hispaniolan Amazon parrots, the opposite is true. This becomes more complicated when comparing fecal CORT or glucocorticoid between sexes, as there is variance in metabolism between sexes, leading to altered values [31]. Altogether, this demonstrates the importance of species- and sex-specific research in psittacines.

Breeding seasons have also been reported to affect CORT concentrations in parrots and other species of birds. In a study examining fecal CORT in captive red-tailed parrots (*Amazona brasiliensis*), it was noted that concentrations were highest in late September, just prior to breeding season, and lowest in May [32]. The results indicated that CORT follows more closely with breeding season, rather than climate, although the two can be closely related. However, other studies have noted the negative effects of CORT and glucocorticoids on reproduction, with male Puerto Rican parrots with higher fecal glucocorticoid concentrations resulting in the paired female producing decreased amounts of both total and fertile eggs [13]. These differences underscore the importance of understanding “normal” variations associated with breeding and how these can have negative effects on individuals. In a study that evaluated seasonal changes of CORT in species of birds living in the Sonoran Desert, some birds breeding in the open desert with restricted access to water were able to suppress their stress response, which reactivated after the breeding season was finished [33]. 

Of significant interest in animal welfare is the stress induced by handling, and in a similar manner, the stress associated with a veterinary visit. Previous studies have demonstrated that with prolonged handling (over an hour time period) in Hispaniolan Amazon parrots (*Amazona ventralis*), there will be an elevation in plasma CORT when compared to the baseline [2]. In another study of the same species, where the handling was intermittent, plasma CORT concentrations increased and plateaued after 20 min. However, “stressful” events in between handling, such as transport, were enough to affect bloodwork findings, namely the heterophil to lymphocyte ratio [16]. In African gray parrots (*Psittacus erithacus*), the heterophil to lymphocyte ratio was also found to correlate with fecal glucocorticoid concentrations [34]. A further study, where sedation with butorphanol and midazolam was given intranasally to Hispaniolan parrots, demonstrated no change in plasma corticosterone in the sedated group versus a control group.

Lastly, but certainly not least important, is the variation that can arise from individual animals. While previous studies across different species have looked at the average among the groups, there can be large interindividual variation [3]. Therefore, as with any test, an individual’s values are best compared with previous results, if possible. While an array of research is available for psittacines and, more broadly, birds, there is still much to be investigated. An area that requires more understanding is the effects of chronic stress on CORT, as no general trend has been established. While it has been suggested that chronic stress can lead to decreased corticosterone concentrations, more research is required [16].

## 3. Current Research

Corticosterone is considered the primary stress hormone in birds. Consequently, one would like to accept the information provided by studies that measure CORT concentrations in birds that are experiencing different environments and conditions. Based on the previously described variables as it relates to measurements of corticosterone concentrations in avian species and the methods used, conclusions must be measured through critical interpretation. When there are conflicting results from studies that measure CORT concentrations, all those involved with the research protocol must be considered. From the manner in which the corticosterone was measured to the techniques used to collect the sample within a given environmental condition. Even with the known inconsistencies between methods used and species differences, research has continued to move forward, which provides an ever-expanding body of knowledge on CORT and its response to stress in birds. In the end, as with most scientific research, the use of CORT to measure stress levels in birds and the best method to measure these levels are still being explored to obtain evidence-based results. Many of the avian research studies involving CORT have looked at the measurements related to how real or perceived stressors affect the overall condition of the bird. The overall condition of the bird may be evaluated as feather quality, immune status, quality of life, metabolic and reproductive activity, and/or response to a typical veterinary clinical setting.

**Figure 3:**
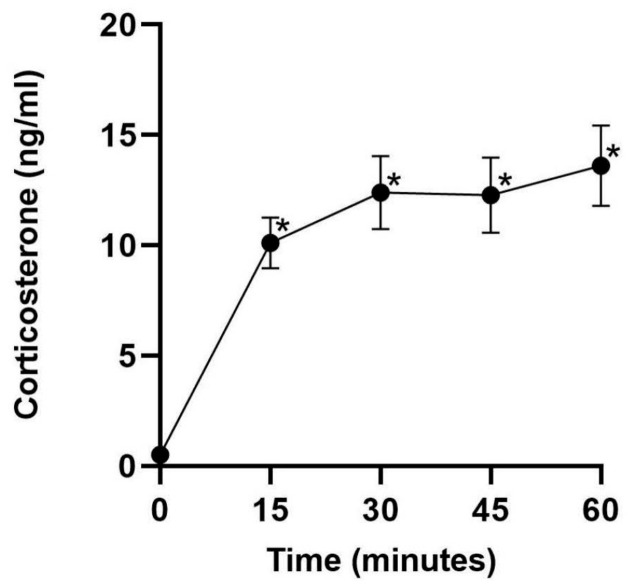
Average corticosterone levels of Hispaniolan parrots (n = 22) were higher after 15 min of restraint compared to baseline (t = 0 min; collected in <3 min) and stayed high for the entire 60 min period. Vertical lines represent the standard error. * Indicates values that significantly differed (*p* < 0.05) from baseline [2].

There are studies that have shown that in stressful conditions, as is typically found in a veterinary hospital, when birds are restrained, stress results in an increased white blood cell count and measured physiological parameters (e.g., cloacal body temperature and respiratory rate) [16,35]. Conversely, another study has reported in poultry that not all stressors have the same effect on white blood cell counts [36]. Based on poultry work, with some extremely stressful conditions, a heteropenia and basophilia occur, suggesting a biphasic leucocyte response [36]. Therein lies the dilemma for veterinarians who treat avian patients and look to determine the effect of stress on their patients. The species differences that may be present and trying to determine the effect of stress on diagnostic test results. With the parrot study that measured both mean CORT concentrations of routine handling, transportation, and blood leukogram counts, all increased over time in the treatment birds [16]. The mean CORT level increased by 60% over the control group but plateaued after 20 min [16]. The baseline CORT was determined by collecting a blood sample from the birds within 3 min without being disturbed for approximately 3 h. Following the initial blood collection, the blood was collected four more times in 20 min intervals to evaluate the white blood cells and CORT concentrations. As stated above, this study found an increase in CORT over the first 20 min, after which the CORT level plateaued [16]. This was also the result in a future study involving the same Hispaniolan Amazon parrots [2]. Another Hispaniolan Amazon parrot study investigated routine handling and restraint on plasma CORT [2]. The difference in this investigation from the study that also evaluated white blood cell responses was how stress was induced; birds were transported to the veterinary hospital, where wing feather trims and claw grooming were performed, as opposed to only being hand-restrained with a towel for an hour. During the travel/grooming study, blood samples were collected at 20 min intervals and with the towel restraint investigation at 15 min intervals. For the towel restraint study, again, the birds’ baseline blood collection was within 3 min of entering the parrot room without prior disturbance for approximately 3 h. The plasma CORT concentrations were measured using enzyme-linked immune assay plates (Arbor Assays; K014-H1/H5). This was different from the travel/grooming study in which the CORT plasma samples were measured using a radioimmunoassay [16]. After 15 min after being restrained by a towel, the plasma CORT concentrations of the parrots significantly increased from approximately 0 ng/mL to 10 ng/mL, and then plateaued over the next three blood collections to just under 15 ng/mL (Figure 3) [2]. Of interest was the evaluation of sex on CORT concentrations in the towel restraint study. When split between male and female parrots, the female parrots’ CORT concentrations were significantly higher than the males from 30 to 60 min while restrained [2]. The difference in CORT concentrations between male and female birds is an area that demands further research. Although the protocol was not set to do a crossover study and specifically look at the sex effect on CORT concentrations while Hispaniolan Amazon parrots were restrained under sedation, a similar trend was noted in another study [37]. A recently completed crossover study with a specific focus on looking at the sex effect on CORT concentrations on restrained sedated Hispaniolan Amazon parrots has recently been completed and results are pending. In the towel restraint study, the CORT concentrations never decreased over time but remained at an elevated level, even at 1 h [2]. The difference in measuring plasma CORT concentrations over fecal and feather analysis is the ability to measure circulating corticosterone concentrations in real time. Moreover, the variability of sample collection and quality is reduced since the blood samples can be collected and processed in a similar manner for each bird throughout a research study. Other variables to consider are when the baseline samples are collected, after the birds have been disturbed and the manner in which the CORT concentrations were measured. Baseline CORT concentrations in the towel restraint study were lower than previously reported [8,16]. Upon examination, explanations include whether the sample was taken 3 min after entering the bird room and method CORT concentrations were analyzed. Also evaluated in the towel restraint study was the CORT concentrations in seven birds exhibiting destructive feather behavior (sample size = 7), measured against those that had unaffected feathers (sample size = 15) [2]. Although CORT concentrations were elevated, there was no significant difference and sex was not factored into the results. The results were similar to a study that evaluated plasma CORT concentrations in African gray parrots [38]. Conversely, other investigations in which fecal glucocorticoid metabolites were evaluated found the concentrations of CORT to be elevated when compared to birds with normal feathering [21,39,40]. The different results between the studies exemplify the need for further understanding of CORT metabolism and how a bird physiologically responds when feather destructive behavior is present.

Hispaniolan Amazon parrots’ plasma CORT concentrations were determined after they had been sedated with midazolam and butorphanol following the baseline blood collection [37]. The sedation study was not a crossover study and birds were separated into control and treatment groups comprising 11 birds each. Based on a previous study, the birds were only sampled twice at 15 and 30 min following the initial blood collection [2]. Results from this investigation found that the plasma CORT concentrations did not differ between the control and treatment groups [37]. The conclusions suggest that even when sedated, the Hispaniolan Amazon parrots elicited a stress response. A second crossover study will reassess the effects of sedation on plasma CORT concentrations while the parrots are restrained.

Stress can be defined as a body’s physiological response to neutralizing antagonistic conditions. For the previously described research, the stressor evaluated has been the trauma of being held, blood collection, and basic grooming procedures. Other studies have looked at environmental conditions and the effect of increased CORT concentrations on birds’ growth and immune system [14,41,42]. The increased research into the effects of stress-induced corticosterone provides promise to enhance the ability of veterinarians to offer better care of both critical and non-critical patients. While a conscious acknowledgement of the risks associated with extrapolating results obtained from one avian species to another is required, the information gained helps promote future studies and serves as a framework for an educated adaptation of this new knowledge. This until more species targeted results are published.

Feather CORT was measured in three groups of greater flamingos with different forms of flight restraint and a control group with no flight restraint [42]. The results revealed no difference between the groups that had their wing feathers trimmed or pinioned and the control, intact, group. This type of study helps zoos and wildlife park personnel determine what effect restricting a bird’s ability to fly within an exhibit has on its stress level and general quality of life.

Other studies have looked at the CORT effects on the overall growth rate and physiological function of birds. Corticosterone concentrations were experimentally increased in house sparrows and were found to be associated with a reduction in feather quality when the birds were molting [40]. Depending on the amount of stress and stage of feather development, “stress bars” in feathers are generally attributed to increased CORT concentrations. Results from an investigation that examined chronic low levels of stress in young American Kestrels found that the control birds had significantly longer wing growth and a reduced level of cutaneous immune function [40]. Chronic stress has not only been found to alter growth and immune function in birds but also tissue-specific CORT receptors in sparrows [41]. For this investigation, brain, internal organs, skin, gastrocnemius muscle and pectoralis muscle CORT receptors were assessed for density in reaction to chronic stress [41]. Only in the pectoralis muscle was a strong effect of chronic stress on the increase in glucocorticoid and mineralocorticoid receptor concentrations [41]. The authors suggest that the increased receptor concentration in the pectoralis muscle may be associated with the use of this muscle as a significant protein reserve when stressed [41].

## 4. Conclusions

As a glucocorticoid hormone released during stressful conditions in birds, CORT is an important factor in the overall well-being and health of birds. Obtaining evidence-based knowledge of what influences the increase in CORT concentrations, the significance of its presence, and determining the detrimental effects of chronic stress will be important to provide better policies for maintaining avian species in zoos, educating companion bird owners of improved husbandry practices, and advancing wildlife conservation efforts that involve avian species. With the technological advances in testing for CORT in avian species, the opportunity to reevaluate previously published work and explore areas of need will provide avenues for a better understanding of this enigmatic hormone. Currently, the astute veterinarian is required to critically assess information published on CORT in avian species and determine how that information is best utilized for the birds in their care.

## Data Availability

The original contributions presented in this study are included in the article. Further inquiries can be directed to the corresponding author.

## References

[1] Olah G., Butchart S.H., Symes A., Guzmán I.M., Cunningham R., Brightsmith D.J., Heinsohn R. (2016). Ecological and socio-economic factors affecting extinction risk in parrots. Biodivers. Conserv..

[2] Parks S.N., Tully T.N., Settle A.L., Lattin C.R. (2023). Handling and restraint induce a significant increase in plasma corticosterone in Hispaniolan Amazon parrots (*Amazona ventralis*). Am. J. Vet. Res..

[3] Cockrem J.F. (2007). Stress, corticosterone responses and avian personalities. J. Ornithol..

[4] Rich E.L., Romero L.M. (2005). Exposure to chronic stress downregulates corticosterone responses to acute stressors. Am. J. Physiol. Regul. Integr. Comp. Physiol..

[5] Xu J., Wang B., Ao H. (2025). Corticosterone effects induced by stress and immunity and inflammation: Mechanisms of communication. Front. Endocrinol..

[6] Reeder D.M., Kramer K.M. (2005). Stress in free-ranging mammals: Integrating physiology, ecology, and natural history. J. Mammal..

[7] Holmes W.N., Phillips J.G., Chester-Jones I., Henderson I. (1976). The adrenal cortex in birds. General and Comparative Endocrinology of the Adrenal Cortex.

[8] Heatley J.J., Oliver J.W., Hosgood G., Columbini S., Tully T.N. (2000). Serum corticosterone concentrations in response to restraint, anesthesia, and skin testing in Hispaniolan Amazon parrots (*Amazona ventralis*). J. Avian Med. Surg..

[9] Romero L.M., Reed J.M. (2005). Collecting baseline corticosterone samples in the field: Is under 3 min good enough?. Comp. Biochem. Physiol. Part A Mol. Integr. Physiol..

[10] Fraisse F., Cockrem J. (2006). Corticosterone and fear behaviour in white and brown caged laying hens. Br. Poult. Sci..

[11] Cockrem J., Silverin B. (2002). Variation within and between birds in corticosterone responses of great tits (*Parus major*). Gen. Comp. Endocrinol..

[12] Silverin B., Arvidsson B., Wingfield J. (1997). The adrenocortical responses to stress in breeding willow warblers Phylloscopus trochilus in Sweden: Effects of latitude and gender. Funct. Ecol..

[13] Ramos-Güivas B., Jawor J.M., Wright T.F. (2021). Seasonal variation in fecal glucocorticoid levels and their relationship to reproductive success in captive populations of an endangered parrot. Diversity.

[14] Butler M.W., Leppert L.L., Dufty A.M. (2010). Effects of small increases in corticosterone levels on morphology, immune function, and feather development. Physiol. Biochem. Zool..

[15] Cabezas S., Carrete M., Tella J.L., Marchant T.A., Bortolotti G.R. (2013). Differences in acute stress responses between wild-caught and captive-bred birds: A physiological mechanism contributing to current avian invasions?. Biol. Invasions.

[16] McRee A.E., Tully T.N., Nevarez J.G., Beaufrere H., Ammersbach M., Gaunt S.D., Fuller R.G., Romero L.M. (2018). Effect of routine handling and transportation on blood leukocyte concentrations and plasma corticosterone in captive Hispaniolan Amazon parrots (*Amazona ventralis*). J. Zoo Wildl. Med..

[17] Davis A., Maney D., Maerz J. (2008). The use of leukocyte profiles to measure stress in vertebrates: A review for ecologists. Funct. Ecol..

[18] Bekhbat M., Glasper E.R., Rowson S.A., Kelly S.D., Neigh G.N. (2018). Measuring corticosterone concentrations over a physiological dynamic range in female rats. Physiol. Behav..

[19] Voit M., Merle R., Baumgartner K., von Fersen L., Reese L., Ladwig-Wiegard M., Will H., Tallo-Parra O., Carbajal A., Lopez-Bejar M. (2020). Validation of an alternative feather sampling method to measure corticosterone. Animals.

[20] Romero L.M., Fairhurst G.D. (2016). Measuring corticosterone in feathers: Strengths, limitations, and suggestions for the future. Comp. Biochem. Physiol. Part A Mol. Integr. Physiol..

[21] Costa P., Macchi E., Valle E., De Marco M., Nucera D.M., Gasco L., Schiavone A. (2016). An association between feather damaging behavior and corticosterone metabolite excretion in captive African grey parrots (*Psittacus erithacus*). PeerJ.

[22] Dudley E., Zhang J., Pahuja H., Narayan E.J. (2024). Determining Physiological Stress Levels of Rescued Birds by Measuring Feather Corticosterone in a Retrospective Study. Preprints. https://www.preprints.org/frontend/manuscript/c785a05fc2746dba8d12928759c3228e/download_pub.

[23] Tempel D.J., Gutiérrez R. (2004). Factors related to fecal corticosterone levels in California spotted owls: Implications for assessing chronic stress. Conserv. Biol..

[24] Arlettaz R., Nusslé S., Baltic M., Vogel P., Palme R., Jenni-Eiermann S., Patthey P., Genoud M. (2015). Disturbance of wildlife by outdoor winter recreation: Allostatic stress response and altered activity–energy budgets. Ecol. Appl..

[25] Lucas J.R., Freeberg T.M., Egbert J., Schwabl H. (2006). Fecal corticosterone, body mass, and caching rates of Carolina chickadees (*Poecile carolinensis*) from disturbed and undisturbed sites. Horm. Behav..

[26] Ebisawa K., Kusuda S., Nakayama S., Pai C., Kinoshita R., Koie H. (2022). Effects of rearing methods on feather-damaging behavior and corticosterone metabolite excretion in the peach-faced lovebird (*Agapornis roseicollis* Vieillot). J. Vet. Behav..

[27] Dehnhard M., Schreer A., Krone O., Jewgenow K., Krause M., Großmann R. (2003). Measurement of plasma corticosterone and fecal glucocorticoid metabolites in the chicken (*Gallus domesticus*), the great cormorant (*Phalacrocorax carbo*), and the goshawk (*Accipiter gentilis*). Gen. Comp. Endocrinol..

[28] Grundei L.-L., Wolf T.E., Brandes F., Schütte K., Freise F., Siebert U., Touma C., Pees M. (2024). Validation of fecal glucocorticoid metabolites as non-invasive markers for monitoring stress in common buzzards (*Buteo buteo*). Animals.

[29] Schwabl P., Bonaccorso E., Goymann W. (2016). Diurnal variation in corticosterone release among wild tropical forest birds. Front. Zool..

[30] Ferreira J.C., Fujihara C.J., Fruhvald E., Trevisol E., Destro F.C., Teixeira C.R., Pantoja J.C., Schmidt E.M., Palme R. (2015). Non-invasive measurement of adrenocortical activity in blue-fronted parrots (*Amazona aestiva*, Linnaeus, 1758). PLoS ONE.

[31] Goymann W. (2012). On the use of non-invasive hormone research in uncontrolled, natural environments: The problem with sex, diet, metabolic rate and the individual. Methods Ecol. Evol..

[32] Popp L.G., Serafini P.P., Reghelin A.L., Spercoski K.M., Roper J.J., Morais R.N. (2008). Annual pattern of fecal corticoid excretion in captive Red-tailed parrots (*Amazona brasiliensis*). J. Comp. Physiol. B.

[33] Wingfield J.C., Vleck C.M., Moore M.C. (1992). Seasonal changes of the adrenocortical response to stress in birds of the Sonoran Desert. J. Exp. Zool..

[34] Bienboire-Frosini C., Alnot-Perronin M., Chabaud C., Asproni P., Lafont-Lecuelle C., Cozzi A., Pageat P. (2018). Assessment of commercially available immunoassays to measure glucocorticoid metabolites in African Grey Parrot (*Psittacus Erithacus*) droppings: A ready tool for non-invasive monitoring of stress. Animals.

[35] Greenacre C.B., Lusby A.L. (2004). Physiologic responses of Amazon parrots (*Amazona species*) to manual restraint. J. Avian Med. Surg..

[36] Maxwell M. (1993). Avian blood leucocyte responses to stress. World’s Poult. Sci. J..

[37] Helms-Pack M.L., Tully T.N., Freeman B.S., Liu C.-C., Stansberry K.R., Tuminello J.A., Lattin C.R. (2025). No Significant Reduction in the Plasma Corticosterone Response of Hispaniolan Amazon Parrots (*Amazona ventralis*) Sedated with Intranasal Midazolam-Butorphanol during Periods of Routine Restraint. J. Zoo Wildl. Med..

[38] Clubb S.L., Cray C., Arheart K.L., Goodman M. (2007). Comparison of selected diagnostic parameters in African grey parrots (*Psittacus erithacus*) with normal plumage and those exhibiting feather damaging behavior. J. Avian Med. Surg..

[39] Owen D., Lane J. (2006). High levels of corticosterone in feather-plucking parrots (*Psittacus erithacus*). Vet. Rec..

[40] Lattin C.R., Reed J.M., DesRochers D.W., Romero L.M. (2011). Elevated corticosterone in feathers correlates with corticosterone-induced decreased feather quality: A validation study. J. Avian Biol..

[41] Lattin C.R., Romero L.M. (2014). Chronic stress alters concentrations of corticosterone receptors in a tissue-specific manner in wild house sparrows (*Passer domesticus*). J. Exp. Biol..

[42] Reese L., Baumgartner K., von Fersen L., Merle R., Ladwig-Wiegard M., Will H., Haase G., Tallo-Parra O., Carbajal A., Lopez-Bejar M. (2020). Feather Corticosterone Measurements of Greater Flamingos Living under Different Forms of Flight Restraint. Animals.

